# The effectiveness and safety of traditional Chinese medicine for the treatment of children with COVID-19

**DOI:** 10.1097/MD.0000000000021247

**Published:** 2020-07-24

**Authors:** Yanqing Li, Lin Bi, Yulin Li, Xiongxin Hu, Quanxi Wang, Xin Liang, Xujun Yu, Liang Dong, Quan Xie

**Affiliations:** a Chengdu University of Traditional Chinese Medicine; b Department of Andrology, The Reproductive and Women-Children Hospital, Chengdu University of Traditional Chinese Medicine; c Hospital of Chengdu University of Traditional Chinese Medicine, Chengdu, Sichuan, P.R. China.

**Keywords:** children, COVID-19, novel coronavirus pneumonia, systematic review, traditional Chinese medicine

## Abstract

**Introduction:**

With the widespread spread of novel coronavirus pneumonia, more and more countries have been affected. Some research reports have shown that traditional Chinese medicine has a significant effect on COVID-19 infection, and the treatment of traditional Chinese medicine is used in some special people, such as children. At present, there is a lack of high-quality systematic reviews on the safety and efficacy of using Chinese medicine to treat children with novel coronavirus pneumonia.

**Materials and methods:**

We will search Cochran library, MEDLINE, EMBASE, China National Knowledge Infrastructure Database (CNKI), China Biomedical Database (CBM), VIP Database (VIP), and Wanfang database for research. This study includes randomized controlled trials (RCTs) and non-RCTs, and uses the Cochrane systematic review to review the safety and efficacy of traditional Chinese medicine in preventing and treating children with novel coronavirus pneumonia. RCT research tools and quantitative research quality assessment tools for non-randomized studies will be used to assess the risk of bias in studies included in the systematic review. We will use Revman 5.3 software for meta-analysis, the main result is odds ratio, and then a subgroup analysis will be performed based on the age, intervention degree, and disease severity of the patients reviewed.

**Ethics and dissemination:**

This systematic review protocol is designed to provide evidence regarding the effectiveness and safety of traditional Chinese medicine for the treatment of children with COVID-19, such evidence may be useful and important for clinical treatment decisions. The results should be disseminated through publication in a peer-reviewed journal. Since the data and results used in the systematic review will be extracted exclusively from published studies, approval from an ethics committee will not be required.

**Registration information:**

PROSPERO CRD42020179150.

## 1 Introduction

Since the outbreak of the novel coronavirus pneumonia in December 2019, many countries have been severely infected and a global pandemic is currently occurring. Globally, as of 2:00 a.m. CEST, April 16, 2020, there have been 1,995,983 confirmed cases of COVID-19, including 131,037 deaths, reported to WHO.^[1]^

So far, there is no special medicine to treat the disease in the world. In our “COVID-19 Treatment Plan (Trial Version 3),” a Chinese medicine treatment plan is proposed, in traditional treatment methods, more than 85% of infected people in China receive traditional Chinese medicine treatment.^[2,3]^ A large number of clinical practice results show that traditional Chinese medicine plays an important role in the treatment of COVID-19.^[4]^

For children, most children have mild clinical manifestations and a good prognosis.^[5]^ Some therapeutic drugs for adult patients are not suitable for children. Therefore, when treating pediatric patients, relatively gentle traditional Chinese medicine treatment can be considered. At present, studies have shown that on the basis of conventional treatment, the combined application of four seasons antiviral drugs is a safe and effective measure for the treatment of respiratory infections in children.^[6]^

Although there are many studies reporting that traditional Chinese medicine treats COVID-19 better, there are fewer reports on children treated with traditional Chinese medicine, and there is a lack of theoretical research and data analysis on more children treated with traditional Chinese medicine. This study aims to collect more clinical data and make a high-quality evaluation of the safety and efficacy of traditional Chinese medicine treatment of children, which is of great significance for pediatricians to make clinical decisions.

## 2 Review objectives

The purpose of this study is to evaluate the efficacy and safety of traditional Chinese medicine in the treatment of children with COVID-19 infection. Due to novel coronavirus pneumonia, novel coronavirus pneumonia is increasing in children all over the world, most of which are mild. Traditional Chinese medicine can effectively improve the symptoms of light patients, so it is necessary to analyze the safety and effectiveness of traditional Chinese medicine treatment for children infected with COVID-19.

## 3 Materials and methods

This is a systematic review and meta-analysis protocol, which is based on the Preferred Reporting Items for Systematic Review and Meta-Analysis Protocols (PRISMA-P) 2015 statement^[7]^ and Cochrane Collaboration Handbook.^[8]^ The data and results used in this paper are form online databases.

### 3.1 Included and excluded criteria

#### 3.1.1 Study design

Randomized controlled trials (RCTs), non-randomized studies and quasi-experimental studies.

#### 3.1.2 Participants

##### 3.1.2.1 Included population

Children and young people (up to 18 years) who have be diagnosed as COVID-19.

##### 3.1.2.2 Excluded population

There is no laboratory evidence of COVID-19 infection in children.

##### 3.1.2.3 Interventions

Traditional Chinese medicine as the main treatment for the intervention measures.

##### 3.1.2.4 Controls

Blank control (no treatment), placebo control (pseudo-drug), cross control (integrated traditional Chinese and western medicine, western medicine treatment) or other active treatment recommended by the guideline.

#### 3.1.3 Outcomes

We will use the following results to evaluate the effectiveness and safety of Chinese medicine treatment.

Main results: effective rate and mortality rate.

Secondary results:

1. Degree of remission of clinical symptoms (fever, diarrhea, nausea, and dyspnea).
2. Time to return to normal for laboratory measures (respiratory rate, oxygen saturation, and lymphocytes).
3. Overall evaluation of lung function after treatment.
4. Proportion of patients admitted to ICU.
5. The frequency at which it needs to be oxygenated.
6. Adverse event rate

### 3.2 Data source

#### 3.2.1 Electronic search database and approach

The databases we will search include Cochran library, EMBASE, MEDLINE, China Biology Medicine Database (CBM), China National Knowledge Infrastructure Database (CNKI), VIP, and Wang Fang database. The search will be started from December 2019 with language restriction in English and Chinese. Our search terms and relative variants will include novel coronavirus pneumonia, new coronavirus, COVID-19, 2019 nCoV, sars-cov-2, child, children, traditional Chinese medicine, Chinese herbal medicine, Chinese patent medicine, Chinese medicine, TCM. The search strategies of MEDLINE are shown in Table 1, and the search strategies of CBM is shown in Table 2.

**Table 1:**
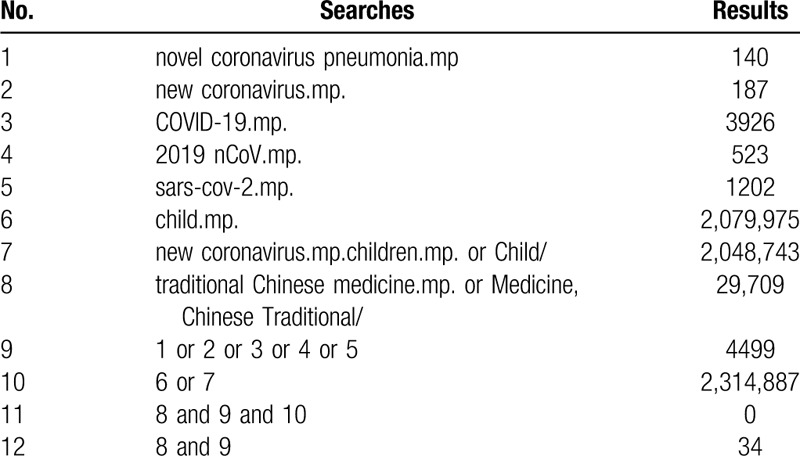
MEDLINE search strategies.

**Table 2:**
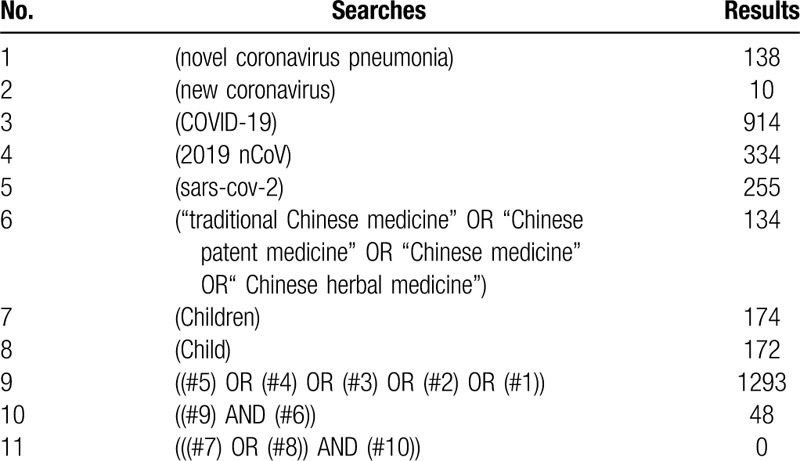
CBM search strategies.

### 3.3 Selection of studies and data extraction

**Figure 1:**
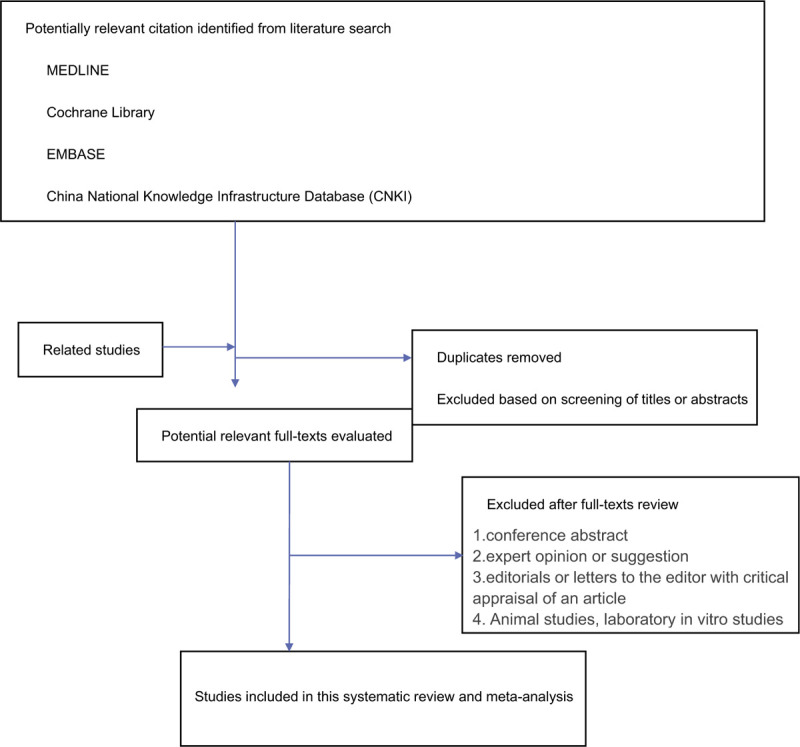
PRISMA flow diagram for identifying, screening and determining the eligibility of and whether to include studies. PRISMA, Preferred Reporting Items for Systematic Review and Meta-Analysis.

Inclusion and exclusion of reference studies will follow PRISMA flow charts to the letter (shown in Fig. 1).

We imported the literature retrieved from each database into the EndnoteX9 software, reviewed and deleted the duplicate literature, and then two reviewers (Xiongxin Hu, Quanxi Wang) screened the title, abstract and other information of the study according to the inclusion and exclusion criteria.

After we get the results, we will download the full text to the corresponding database according to the results, and then the other two reviewers (Jiaying Li, Lin Bi) will read the full text and filter it again. When the literature results are repeated, the study with large sample content or long follow-up time will be selected. If the repeated study cannot be determined, the author of this study will be contacted. Any inconsistencies in the process will be resolved by discussion.

A unified data extraction form (excel spreadsheet) will be generated after the review panel discussion. The data extraction form contains the following information:

Research characteristics: publishing time, publishing unit, publishing country, research object, research type, and research method.

Patient baseline characteristics, exposure characteristics, intervention measures (treatment method, administration route, and dosage) and outcome indicators; Information such as risk assessment of bias. In the process of data extraction, if there is any disagreement between the two reviewers, the third reviewer will be contacted for discussion and resolution.

After data extraction, we will use the Cochrane study tool to assess the quality of the study and the risk of bias. The two reviewers will use the double-blind method to independently assess the risk of bias. If there is any difference, it will be resolved through discussion.

### 3.4 Risk of bias assessment

We will use the Cochrane bias risk assessment tools from random sequence and allocation concealment, blinding researchers and subjects, study outcome blind evaluation, integrity of outcome data, selective reporting of research findings, and other sources of bias that six aspects to evaluate risk of bias, according to the risk of bias assessment criteria to make a “low risk of bias,” “high risk of bias,” and “not clear” to judge the results.

### 3.5 Data analysis and synthesis

Meta analysis is carried out by using RevMan 5.3, a standardized software for systematic evaluation by Cochrane collaboration network. *Q* test is used to determine the heterogeneity of the data. *I*^2^ index is used to describe the percentage of heterogeneity in the total variation caused by non-sampling error caused by each study. If *I*^2^ < 50%, it is considered that there is no obvious heterogeneity of the data. If the test results indicate statistical heterogeneity, the source of heterogeneity needs to be further analyzed. According to the heterogeneity size, it is necessary to determine whether the data can be analyzed by using the random effect model directly. If the heterogeneity is small, the effect scale can be merged by using the random effect model directly. If it is larger, it is necessary to adopt subgroup analysis to exclude the sources of heterogeneity, and then merge, and then use forest map to reflect the merger results. Generally, *I*^2^ ≥ 75% is regarded as the critical point of high heterogeneity. For categorical variables, we will use odds ratio (OR) and relative risk (RR) as the effect scale. For the data of quantitative variables, the weighted mean difference (WMD) and the standardized mean difference (SMD) are used as the effect scales. These are all expressed as 95% confidence intervals. Funnel plot and Egger linear regression method are used to judge whether there is publication bias. If *P* value < .10, there is publication bias.

### 3.6 Subgroup analysis and sensitivity analysis

When the data is collected, if it can be allowed for subgroup analyses, we will analyze it according to the age of the patients, the degree of intervention, and disease severity. If the interaction test between related subgroups is <0.05, we will analyze and adjust them to eliminate deviation and risk to ensure the stability of evidence.

### 3.7 Sensitivity analysis

After excluding the abnormal results, the meta-analysis was performed again and the results of two meta-analyses were compared to explore the stability of the results.

### 3.8 Publication bias

Published bias will be measured by using a funnel plot (by Review Manager 5.3 software), Begg test and Egger test (by Stata software 14.0).^[9,10]^

## 4 Ethics and dissemination

The purpose of this system review is to provide evidence for the efficacy and safety of traditional Chinese medicine for children with COVID-19. These evidences are of great significance in providing clinical treatment decisions. The results of the study will be published in public journals. Because the data and results used in this systematic review are all from published studies, approval from an ethics committee is not required.

## 5 Discussion

At present, there is no effective treatment for patients infected with COVID-19, so the morbidity and mortality are higher than influenza. Traditional Chinese medicine has been widely used in clinical practice in China, but in many countries in the world, it is still challenging for traditional Chinese medicine to treat patients with COVID-19 infection.^[11]^ During the outbreak of severe acute respiratory syndrome (SARS) in 2003, the effectiveness of traditional Chinese medicine in controlling infectious diseases was confirmed. Therefore, the Chinese government encourages the use of herbal medicine to combat this new type of viral pneumonia.^[12]^ According to reports, 26 Chinese herbal medicines have been screened and considered to be effective for COVID-19. They can directly inhibit novel coronavirus pneumonia.^[12]^ China has launched 303 clinical trials aimed at evaluating the treatment effect and safety of patients. Among them, 50 cases (16.5%) were related to the application of traditional Chinese medicine.

Depending on the stage of the disease and symptoms, different herbs used in the Chinese medicine system are recommended for the treatment of COVID-19. During clinical treatment, Qingfei Paidu Decoction, Xiyanping Injection, Xuebijing Injection, Reduning Injection, and Tanreqing Injection,^[2]^ among them, yinqiao powder is used for patients with high fever and Sangju drink is used for patients with severe cough, which can clear lung heat, remove phlegm, stop cough, regulate lung, and restore normal lung function.^[11]^

Recently, 4 provincial hospitals in China applied Qingfei Paidu Decoction to treat 214 patients with COVID-19. The course of treatment was 3 days. The total effective rate was more than 90%. Among them, 60% of patients had significantly improved symptoms and imaging performance, and 30% of patients symptoms are stable without deterioration. At the Eighth People's Hospital in Guangzhou, China, doctors found that 50 patients with mild COVID-19 were treated with Toujie Quwen granule. Their overall symptoms improved significantly without any serious symptoms. The study found that this drug can significantly improve the clinical symptoms caused by COVID-19, and has a tendency to reduce the incidence of severe pneumonia.^[13]^

In view of the fact that Chinese medicine treatment of COVID-19 infection in adults can effectively improve the clinical symptoms of mild patients, it may be considered to apply Chinese medicine treatment to children infected with COVID-19, so that the children's recovery is good. According to the latest diagnosis and treatment plan, the symptoms of children's cases are relatively mild, so it is very necessary to apply Chinese medicine treatment to the treatment of children infected.^[14]^

At present, there are limited clinical trial data on the effectiveness and safety of traditional Chinese medicine treatment for infected children, and there is a lack of a comprehensive review and summary. Therefore, this article aims to study the Chinese medicine treatment of COVID-19 infected children and provide clinicians with decision-making for clinical diagnosis and treatment of COVID-19 infection in children. The systematic review also has certain limitations. First, the literature we included only included Chinese and English literature, which may cause some omissions. Secondly, the included studies were non-RCTs and observational studies, which may also lead to lack of reliability in the conclusions.

## Author contributions

All authors have read and approved the manuscript, agreed to publish it, and made sure it is true.

LYQ, YXJ, DL, XQ provide COVID-19 related professional knowledge.

LYL, BL, HXX undertake data management.

LYQ, BL, WQX complete the draft writing.

LYQ, LYL, BL complete the revision and editing.

LYL, BL, WQX provide research methods.

LX manages the project.

Research design and concept of XQ and LYQ.

Resources provided by LX, YXJ, LYQ, XQ.

LX, XQ, LYQ reviewed and approved the release.

LYL, BL, HXX, WQX carry out software operation.
